# Brain-derived neurotrophic factor-TrkB signaling in the medial prefrontal cortex plays a role in the anhedonia-like phenotype after spared nerve injury

**DOI:** 10.1007/s00406-018-0909-z

**Published:** 2018-06-07

**Authors:** Xi Fang, Chun Yang, Shan Li, Gaofeng Zhan, Jie Zhang, Niannian Huang, Xiangxi Du, Hui Xu, Kenji Hashimoto, Ailin Luo

**Affiliations:** 1 Department of Anesthesiology, Tongji Hospital, Tongji Medical College, Huazhong University of Science and Technology, No. 1095 Jiefang Avenue, Wuhan, 430030 China; 2 Division of Clinical Neuroscience, Chiba University Center for Forensic Mental Health, Chiba, 260-8670 Japan

**Keywords:** Anhedonia, BDNF, Individual differences, Neuropathic pain, TrkB

## Abstract

Although depressive symptoms including anhedonia (i.e., loss of pleasure) frequently accompany pain, little is known about the risk factors contributing to individual differences in pain-induced anhedonia. In this study, we examined if signaling of brain-derived neurotrophic factor (BDNF) and its receptor tropomyosin-receptor-kinase B (TrkB) contribute to individual differences in the development of neuropathic pain-induced anhedonia. Rats were randomly subjected to spared nerved ligation (SNI) or sham surgery. The SNI rats were divided into two groups based on the results of a sucrose preference test. Rats with anhedonia-like phenotype displayed lower tissue levels of BDNF in the medial prefrontal cortex (mPFC) compared with rats without anhedonia-like phenotype and sham-operated rats. In contrast, tissue levels of BDNF in the nucleus accumbens (NAc) of rats with an anhedonia-like phenotype were higher compared with those of rats without anhedonia-like phenotype and sham-operated rats. Furthermore, tissue levels of BDNF in the hippocampus, L2–5 spinal cord, muscle, and liver from both rats with or without anhedonia-like phenotype were lower compared with those of sham-operated rats. A single injection of 7,8-dihydroxyflavone (10 mg/kg; TrkB agonist), but not ANA-12 (0.5 mg/kg; TrkB antagonist), ameliorated reduced sucrose preference and reduced BDNF-TrkB signaling in the mPFC in the rats with anhedonia-like phenotype. These findings suggest that reduced BDNF-TrkB signaling in the mPFC might contribute to neuropathic pain-induced anhedonia, and that TrkB agonists could be potential therapeutic drugs for pain-induced anhedonia.

## Introduction

Pain is one of the most common ailments that make patients seek medical treatment, representing a major clinical, social, and economic problem. Pain and depression frequently manifest together. Epidemiological studies have shown that the prevalence of depression in patients with pain is higher compared with that when these two conditions are separately evaluated [1–3]. Approximately 30% of patients in pain experience depression [4, 5]. A recent longitudinal analysis demonstrated that chronic pain strongly predicts the development of more depressive symptoms in patients with pain compared with those without pain [6]. These epidemiological studies suggest that individual differences exist in the development of pain and depression comorbidities [1, 2, 7–9]. Although pain and depression share biological pathways, the precise mechanisms underlying the comorbidity of pain and depression remain unknown. In addition, the possible predisposing factors for individual differences in these comorbidities remain poorly understood.

The signaling pathway comprising brain-derived neurotrophic factor (BDNF) and its specific receptor tropomyosin-receptor-kinase B (TrkB) plays a key role in the pathophysiology of depression and in the antidepressant functions of antidepressants [10–18]. Alterations in BDNF-TrkB signaling in the brain have been implicated in the pathogenesis of depression and antidepressant mechanisms. Decreased BDNF-TrkB signaling in the prefrontal cortex (PFC) and hippocampus was shown in conditions of chronic unpredictable mild stress (CUMS) [19], inflammation [20–22], chronic social defeat stress (CSDS) [23–25], and learned helplessness (LH) [26, 27]. In contrast, increased BDNF-TrkB signaling in the nucleus accumbens (NAc) was detected in inflammation [20, 21], CSDS [23–25], and LH models [26, 27]. Recent studies suggest that regional differences in BDNF-TrkB signaling confer resilience to inescapable stress [27, 28]. Accumulating evidence suggests that BDNF-TrkB signaling also plays a crucial role in pain [29, 30]. A recent study showed that alterations in inflammatory cytokines and BDNF might contribute to neuropathic pain-induced depression [31]. However, little is known about the detailed role of BDNF-TrkB signaling in individual emotional responses to peripheral nerve injury.

According to the Diagnostic and Statistical Manual of Mental Disorders (DSM-5), the two core symptoms of depression are depressed mood and anhedonia. It is important to investigate the role of anhedonia in pain, because it specifically predicts worse treatment outcome [32] and a longer and more severe course of depression [33]. The purpose of the current study was to examine whether BDNF-TrkB signaling might contribute to the individual differences in anhedonia-like phenotype in rats after spared nerve injury (SNI). Furthermore, we examined if 7,8-dihydroxyflavone (7,8-DHF; a TrkB agonist) [20, 26, 34] or ANA-12 (a TrkB antagonist) [20, 26, 35, 36] could improve anhedonia-like symptoms and alterations in BDNF-TrkB signaling in anhedonia-susceptible rats after SNI.

## Materials and methods

### Animals

Male Sprague Dawley (SD) rats (weighing 180–230 g) were purchased from the Laboratory Animal Centre of Tongji Medical College, Huazhong University of Science and Technology (Wuhan, China). The animals were housed under 12 h light/dark cycle with free access to food and water. Procedures of this animal experiment were in accordance with the National Institute of Health Guide for the Care and Use of Laboratory Animals. The experimental protocols were approved by the Experimental Animal Committee of Tongji Hospital, Tongji Medical College, Huazhong University of Science and Technology.

### Experimental design

**Fig. 1:**
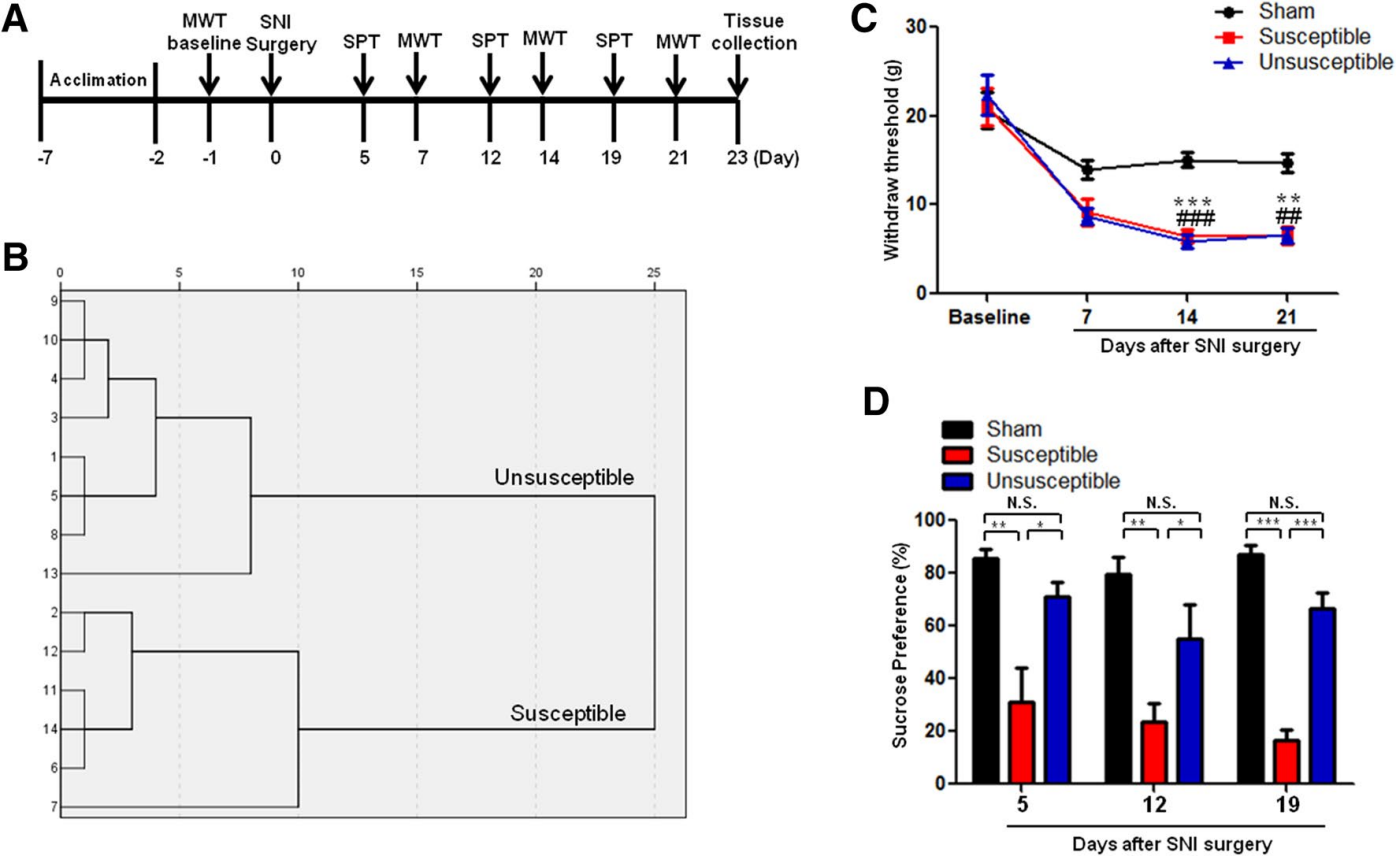
Comparisons of MWT and SPT in sham, anhedonia susceptible, and unsusceptible rats. **a** Schedule of SNI, MWT, and SPT. SNI was performed on day 0 after 7 day acclimation. MWT was measured on days 7, 14, and 21 after SNI, respectively. SPT was performed on days 5, 12, and 19 after SNI, respectively. **b** Dendrogram of hierarchical clustering analysis. A total of 14 SNI rats were divided into anhedonia susceptible and unsusceptible groups by SPT results of hierarchical clustering analysis. **c** MWT (time: *F*_(3,15)_ = 93.989, *P* < 0.001; Group: *F*_(2,10)_ = 14.526, *P* = 0.001; interaction: *F*_(6,30)_ = 3.974, *P* = 0.005) was measured on days 7, 14, and 21 in the sham, anhedonia unsusceptible, and anhedonia susceptible groups after SNI, respectively. Data are shown as mean ± S.E.M. (*n* = 6–8). **P < 0.01 or ***P < 0.001, susceptible group vs sham group. ^##^*P* < 0.01 or ^###^*P* < 0.001, unsusceptible group vs sham group. **d** SPT (time: *F*_(2,10)_ = 2.723, *P* = 0.114; Group: *F*_(2, 10)_ = 51.176, *P* < 0.001; interaction: *F*_(4,20)_ = 0.424, *P* = 0.790) was measured in the sham, anhedonia unsusceptible, and anhedonia susceptible groups on days 5, 12, and 19 after SNI, respectively. Data are shown as mean ± S.E.M. (*n* = 6–8). ***P < 0.05, **P < 0.01 or ***P < 0.001. *MWT* mechanical withdrawal test, *N.S*. not significant, *SNI* spared nerve injury, *SPT* sucrose preference test

As shown in Fig. 1a, rats were acclimated to environment for 6 days. Then, the mechanical withdrawal threshold (MWT) was performed 1 day before the spared nerve injury (SNI) surgery for baseline measurement. MWT was implemented from days 6, 12, and 19 after surgery. Sucrose preference test (SPT) was implemented from days 7, 14, and 21 after surgery. 23 days after SNI surgery, medial prefrontal cortex (mPFC), hippocampus and nucleus accumbens (NAc) of brain, liver, muscle, and L2-5 spinal cord tissues were collected. In addition, in Fig. 5a, anhedonia-susceptible rats were intraperitoneally injected with either 7,8-dihydroxyflavone (7,8-DHF, a TrkB agonist) or ANA-12, (N2-(2-{[(2-oxoazepan-3-yl) amino]carbonyl}phenyl)benzo[b]thiophene-2-carboxamide, a TrkB antagonist). 7,8-DHF (Catalog number: ab120996, Abcam, UK, 10 mg/kg), and ANA-12 (Catalog number: HY-12497, MedChem Express, USA, 0.5 mg/kg) were prepared in vehicle of 17% dimethyl sulfoxide (DMSO) in phosphate-buffered saline. The SPT and MWT were performed on days 12 and 14, respectively. On day 15, mPFC and NAc of rats were collected for measurement.

### SNI

The SNI surgery was performed as previously described [37]. Rats were anesthetized with 10% chloral hydrate (3 ml/kg) and then the skin of left thigh was incised. The sciatic nerve and its three terminal branches after bluntly dissecting biceps femoris muscle were totally exposed. The common peroneal and tibial nerves were ligated with a 4–0 silk and cut off the distal to the ligation. The muscle and skin were sutured with a 4–0 silk. Rats in the sham group were exposed to the sciatic nerve and its three terminal branches but without ligated, and cut off the common peroneal and tibial nerves.

### MWT

Before MWT, rats were placed in plexiglass chambers with a wire net floor for 30 min avoiding the stress resulting from the test conditions. The Electronic Von Frey (UGO BASILE S.R.L., Italy) filaments were applied to the lateral 1/3 of right paws. The paws’ quick withdrawal or flinching was considered as a positive response. Every filament stimuli were applied four times with a period of 30 s interval.

### SPT

Rats were exposed to water and 1% sucrose solution for 48 h, followed by 24 h of water and food deprivation and a 24 h exposure to two identical bottles, one is water, and another is 1% sucrose solution. The bottles containing water and sucrose were weighed before and at the end of this period and the sucrose preference was determined.

### Western blot

Samples were homogenized with RIPA buffer (150 mM sodium chloride, Triton X-100, 0.5% sodium deoxycholate, 0.1% sodium dodecyl sulfate, 50 mM Tris, and pH 8.0) at 4 °C for 30 min, and were then centrifuged for 15 min at 4 °C. BCA protein assay kit (Boster, Wuhan, China) was used to determine the protein levels in supernatant. The samples were separated by 10% sodium dodecyl sulfate–polyacrylamide gel electrophoresis and were transferred to polyvinylidene fluoride membranes (Millipore, Bedford, MA, USA). Bands were blocked with 5% BSA in TBST (0.1%Tween 20 in Tris-buffered saline) for 1 h at room temperature. Relative primary antibodies were incubated at 4 °C overnight: rabbit mature BDNF (1:500, Sangon Biotech, Shanghai, China), rabbit proBDNF (1:500; Origene, Rockville, MD, USA), rabbit TrkB (1:1000, Cell Signaling Technology, Danvers, MA, USA), rabbit phosphorylated p-TrkB (1:1000, Affinity, Cincinnati, OH, USA), and mouse GAPDH (1:1000, Qidongzi, Wuhan, China). Then, bands were washed with TBST and incubated second antibody for 2 h at room temperature: goat anti-rabbit IgG horseradish peroxidase or goat anti-rabbit IgG horseradish peroxidase (1:5000, Qidongzi, Wuhan, China). Finally, these bands were detected by enhanced chemiluminescence reagents (Qidongzi, Wuhan, China) with the ChemiDocXRS chemiluminescence imaging system (Bio-Rad, Hercules, CA, USA).

### Statistical analyses

The data show as the mean ± standard error of the mean (SEM). Analysis was performed using PASW Statistics 20 (formerly SPSS Statistics; SPSS). Comparisons between groups were performed using the one-way analysis of variance (ANOVA) or two-way ANOVA, followed by post hoc Tukey test. Hierarchical cluster analysis of SPT was applied to classify the anhedonia susceptible or unsusceptible rats. The *P* values of less than 0.05 were considered statistically significant.

## Results

### The results of WMT and SPT among the sham, anhedonia susceptible, and unsusceptible rats

Anhedonia susceptible and unsusceptible rats were divided by hierarchical cluster analysis of sucrose preference test (Fig. 1b). WMT was significantly decreased in both anhedonia susceptible and unsusceptible rats as compared with that of sham on days 7, 14, and 21 after SNI surgery (Fig. 1c). However, there was no any change in the WMT between anhedonia susceptible and unsusceptible rats, although WMT in the both groups was significantly lower than sham group (Fig. 1c). Furthermore, anhedonia susceptible rats showed a significant decrease in SPT as compared with those in the sham and unsusceptible rats on days 5, 12, and 19 after SNI, respectively (Fig. 1d).

### Alterations in the tissue levels of BDNF in selected tissues

**Fig. 2:**
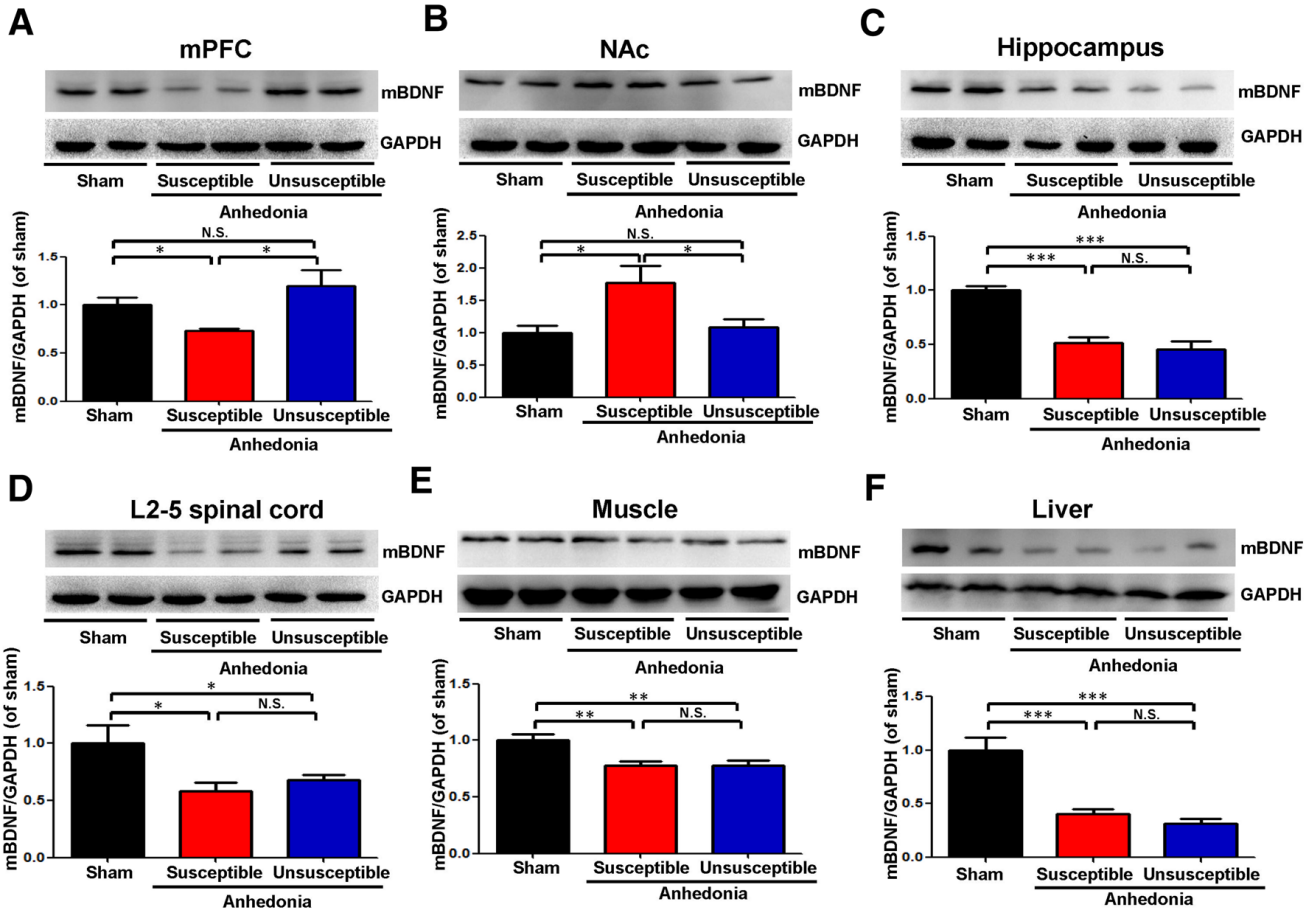
Levels of BDNF in selected tissues among the sham, anhedonia susceptible and unsusceptible groups. **a** mPFC (*F*_(2,15)_ = 6.108, *P* = 0.011). **b** NAc (*F*_(2,15)_ = 5.8, *P* = 0.014). **c** Hippocampus (*F*_(2,15)_ = 59.856, *P* < 0.001); **d** L2–5 spinal cord (*F*_(2,15)_ = 4.307, *P* = 0.033). **e** Muscle (*F*_(2,15)_ = 8.166, *P* = 0.004). **f** Liver (*F*_(2,15)_ = 28.928, *P* < 0.001). Data are shown as mean ± S.E.M. (*n* = 6). *P < 0.05, **P < 0.01 or ***P < 0.01. *BDNF* brain-derived neurotrophic factor, *mPFC* medial prefrontal cortex, *NAc* nucleus accumbens, *N.S*. not significant

As shown in Fig. 2, there were alterations in the BDNF levels in mPFC, NAc, hippocampus, L2–5 spinal cord, muscle, and liver in the rats with SNI-induced anhedonia. Post hoc analysis showed that anhedonia susceptible rats significantly lower levels of mature BDNF in the mPFC than those in sham or anhedonia unsusceptible groups (Fig. 2a). In contrast, anhedonia susceptible rats demonstrated significant higher levels of mature BDNF in the NAc than those in sham or unsusceptible groups. Furthermore, tissue levels of mature BDNF in the L2-5 spinal cord, muscle, and liver of anhedonia susceptible and unsusceptible rats were significantly lower than those on sham group (Fig. 2d–f).

### Alterations in the tissue levels of proBDNF in selected tissues

**Fig. 3:**
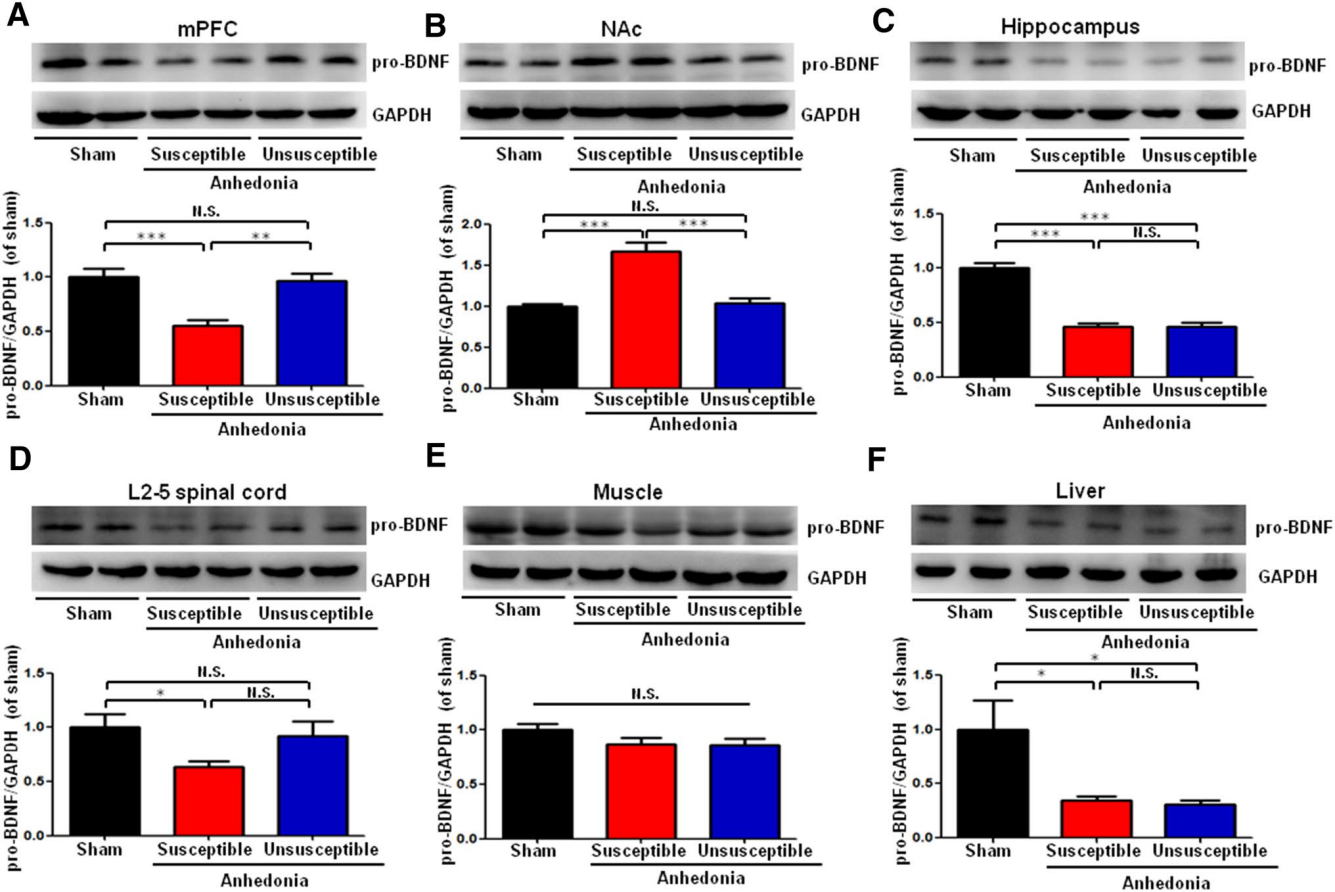
Levels of proBDNF in selected tissues among the sham, anhedonia susceptible, and unsusceptible groups. **a** mPFC (*F*_(2,15)_ = 15.179, *P* = 0.001). **b** NAc (*F*_(2,15)_ = 24.61, *P* < 0.001). **c** Hippocampus (*F*_(2,15)_ = 59.837, *P* < 0.001). **d** L2–5 spinal cord (*F*_(2,15)_ = 3.021, *P* = 0.079). **e** Muscle (*F*_(2,15)_ = 2.466, *P* = 0.119). **f** Liver (*F*_(2,15)_ = 6.11, *P* = 0.031). Data are shown as mean ± S.E.M. (*n* = 6). *P < 0.05, **P < 0.01 or ***P < 0.01. *proBDNF* precursor of BDNF, *mPFC* medial prefrontal cortex, *NAc* nucleus accumbens, *N.S*. not significant

Mature BDNF is prepared from its precursor proBDNF. Tissue levels of proBDNF were significantly lower in the mPFC and L2–5 spinal cord of anhedonia susceptible rats compared sham or unsusceptible groups (Fig. 3a, d). In contrast, tissue levels of proBDNF in the NAc from anhedonia susceptible rats were significantly higher than those of sham or unsusceptible rats. Tissue levels of proBDNF in the hippocampus and liver from anhedonia susceptible rats were significantly lower than those of sham rats, although there was no change between anhedonia susceptible rats and unsusceptible rats (Fig. 3c, f). Furthermore, there was no difference in proBDNF in the muscle among three groups (Fig. 3e).

### Alterations in phosphorylated p-TrkB and TrkB levels in the selected tissues

**Fig. 4:**
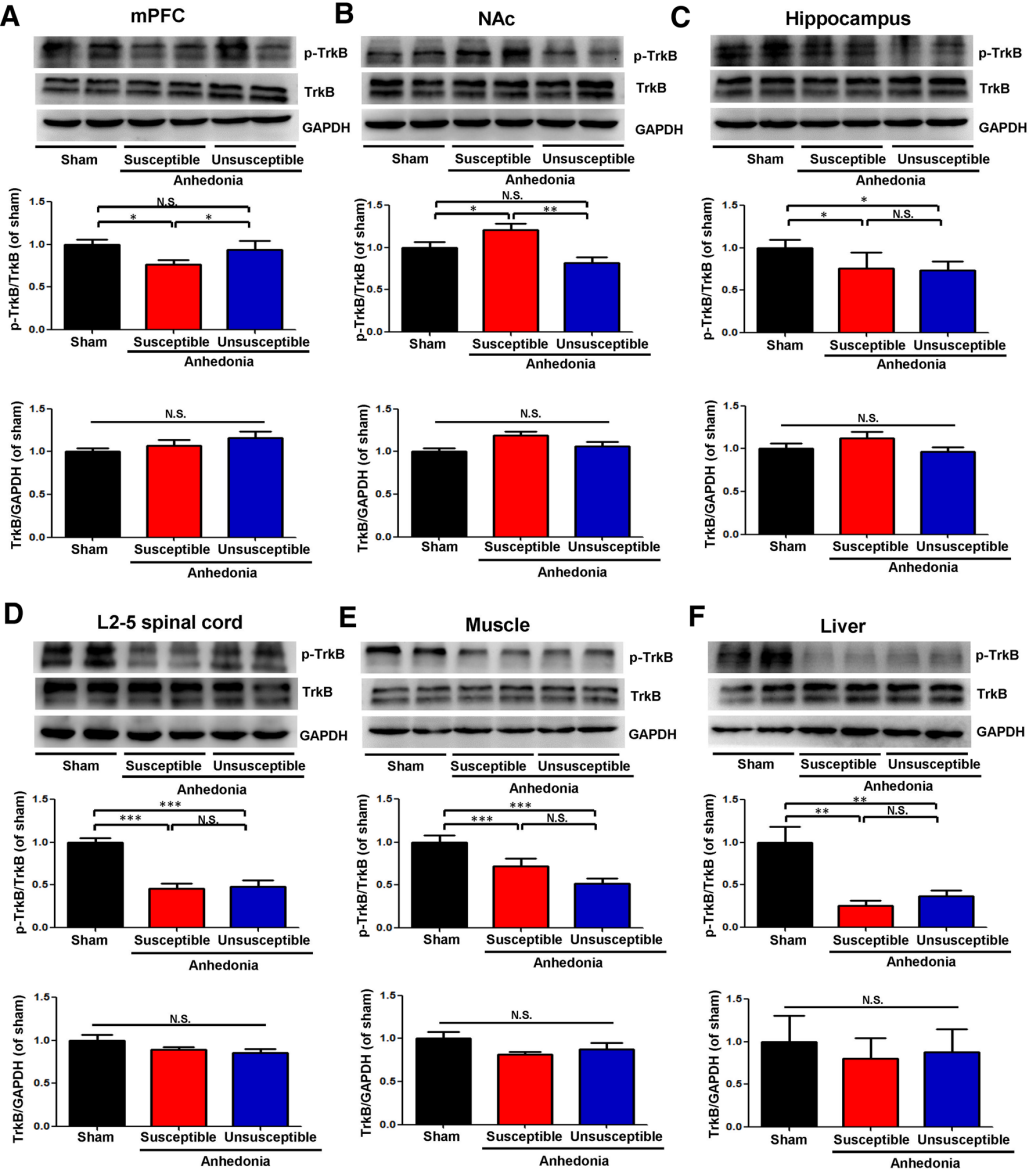
Levels of p-TrkB and TrkB in selected tissues among the sham, anhedonia susceptible, and unsusceptible groups. **a** p-TrkB/TrkB ratio (*F*_(2,15)_ = 6.994, *P* = 0.027) and TrkB (*F*_(2,15)_ = 1.565, *P* = 0.241) levels in the mPFC. **b** p-TrkB/TrkB ratio (*F*_(2,15)_ = 10.551, *P* = 0.002) and TrkB (*F*_2,15_=1.729, *P* = 0.211) levels in the NAc; **c** p-TrkB/TrkB ratio (*F*_(2,15)_ = 8.163, *P* = 0.018) and TrkB (*F*_(2,15)_ = 1.67, *P* = 0.221) levels in the hippocampus. **d** p-TrkB/TrkB ratio (*F*_(2,15)_ = 24.098, *P* < 0.001) and TrkB (*F*_(2,15)_ = 2.376, *P* = 0.127) levels in the L2–5 spinal cord. **e** p-TrkB/TrkB ratio (*F*_(2,15)_ = 9.747, *P* = 0.002) and TrkB (*F*_(2,15)_ = 2.133, *P* = 0.153) levels in the muscle. **f** p-TrkB/TrkB ratio (*F*_(2,15)_ = 11.089, *P* = 0.001) and TrkB (*F*_(2,15)_ = 0.13, *P* = 0.879) levels in the liver. Data are shown as mean ± S.E.M. (*n* = 6). *P < 0.05, **P < 0.01 or ***P < 0.001. *mPFC* medial prefrontal cortex, *NAc* nucleus accumbens, *N.S*. not significant, *p-TrkB* phosphorylation-tropomyosin-receptor kinase B

To clarify whether TrkB activation or inhibition underpins anhedonia-like behavior in SNI rats, we performed immunoblot analyses of TrkB and phosphorylated TrkB (p-TrkB), an activated form of TrkB, in samples from the mPFC, NAc, hippocampus, L2–5 spinal cord, muscle, and liver. The anhedonia susceptible rats, but not unsusceptible rats, had lower p-TrkB/TrkB ratio in the mPFC (Fig. 4a). In addition, both anhedonia susceptible and unsusceptible rats had lower p-TrkB/TrkB ratio in the hippocampus, L2–5 spinal cord, muscle, and liver (Fig. 4c–f). In contrast, p-TrkB/TrkB ratio in the NAc in the anhedonia susceptible rats was significantly higher than those of sham rats or anhedonia unsusceptible rats (Fig. 4b). However, there were no significant changes of total TrkB levels in the all regions among the three groups.

### Effects of 7,8-DHF and ANA-12 on the behavioral abnormalities in anhedonia susceptible rats after SNI

**Fig. 5:**
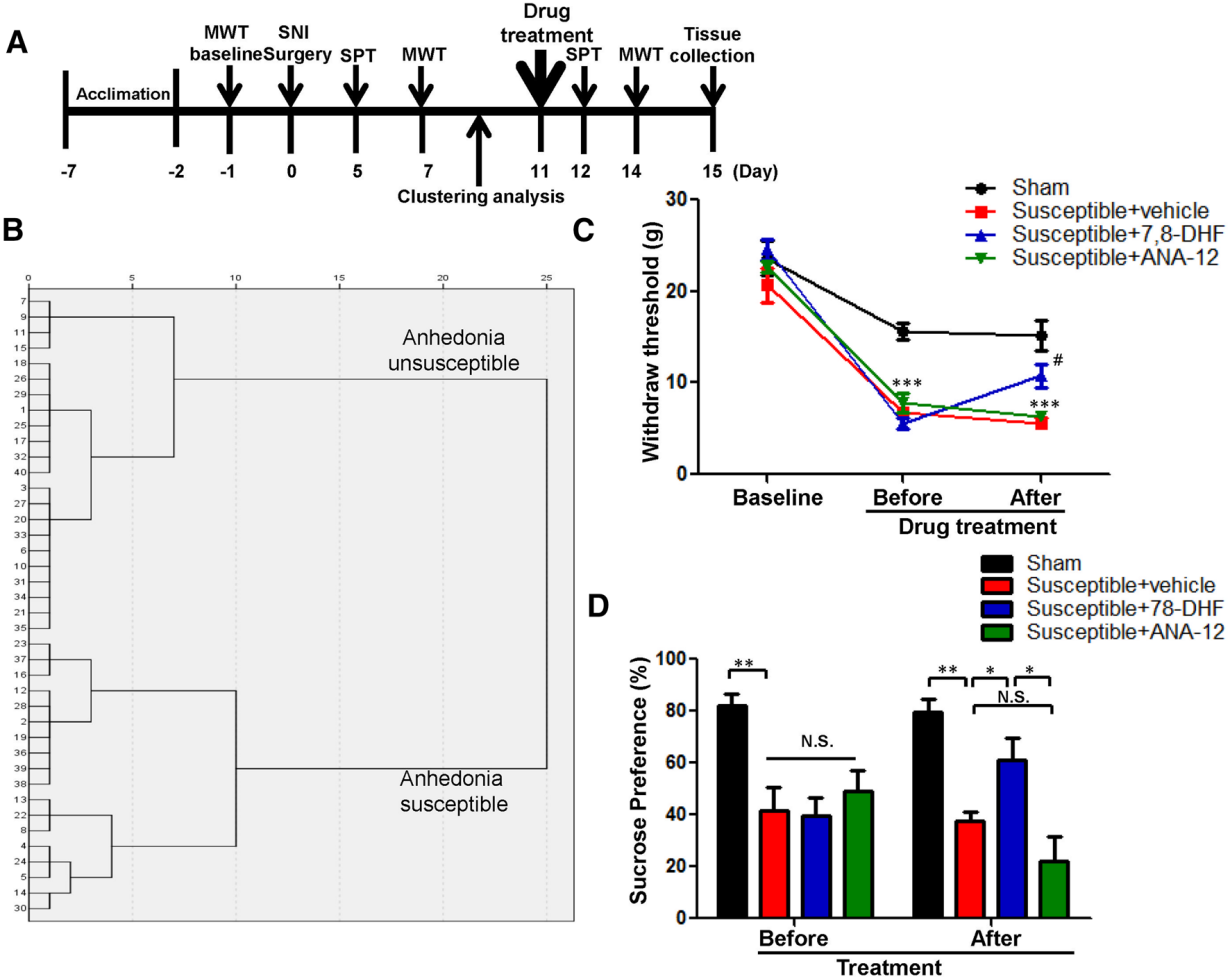
Effects of 7,8-DHF and ANA-12 on behavioral abnormalities in anhedonia susceptible rats. **a** Schedule of SNI, MWT, and SPT. SNI was performed 7 day acclimation. MWT was measured on days 7 and 14 after SNI, respectively. SPT was performed on days 5 and 12 after SNI, respectively. Vehicle (1 ml/kg), 7,8-DHF (10 mg/kg), or ANA-12 (0.5 mg/kg) was intraperitoneally injected at a single dose on day 8. **b** Dendrogram of hierarchical clustering analysis. A total of 40 SNI rats were divided into anhedonia susceptible and unsusceptible groups by SPT results of hierarchical clustering analysis. **c** MWT (time: *F*_(2,10)_ = 239.822, *P* < 0.001; Group: *F*_(3,15)_ = 20.158, *P* < 0.001; interaction: *F*_(6,30)_ = 7.021, *P* < 0.001). ***P < 0.001 v.s. sham group; ^#^*P* < 0.05 v.s. susceptible + vehicle group. **d** SPT (time: *F*_(1,5)_ = 0.260, *P* = 0.632; Group: *F*_(3,15)_ = 11.040, *P* < 0.001; interaction: *F*_(3,15)_ = 5.924, *P* = 0.007). *P < 0.05, **P < 0.01 or ***P < 0.001. Data are shown as mean ± S.E.M. (*n* = 6). *N.S*. not significant, *SNI* spared nerve injury, *SPT* sucrose preference test, *MWT* mechanical withdrawal test

Eighteen anhedonia susceptible rats were selected after hierarchical cluster analysis of SPT for further study (Fig. 5a, b). Anhedonia susceptible rats were treated with a single dose of 7,8-DHF (10 mg/kg) or ANA-12 (0.5 mg/kg). 7,8-DHF significantly restored SNI-induced decrease in scores of MWT and SPT, while ANA-12 did not elicit these improving effects (Fig. 5c, d). The data suggest that 7,8-DHF, but not ANA-12, has anti-anhedonia effects in the anhedonia susceptible rats after SNI.

### Effects of 7,8-DHF and ANA-12 on the alterations in the BDNF-TrkB signaling in the mPFC and NAc of anhedonia susceptible rats

**Fig. 6:**
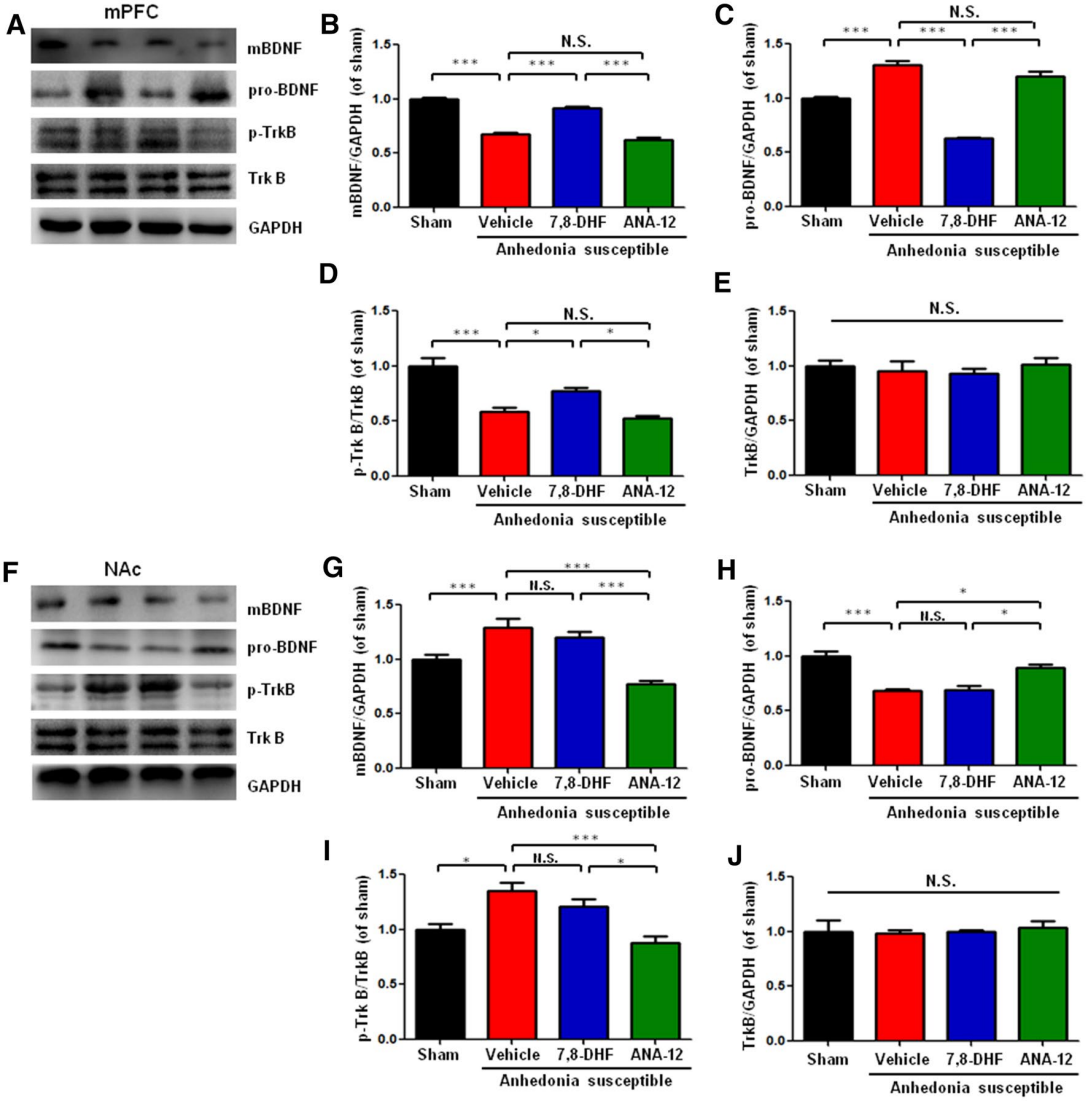
Effects of 7,8-DHF and ANA-12 on BDNF-TrkB signaling in mPFC and NAc in anhedonia susceptible rats. **a** Western blot bands of BDNF-TrkB signaling in the mPFC. **b** BDNF level in the mPFC (*F*_(3,20)_ = 119.273, *P* < 0.001). **c** proBDNF level in the mPFC (*F*_(3,20)_ = 99.914, *P* < 0.001). **d** p-TrkB/TrkB ratio in the mPFC (*F*_(3,20)_ = 22.731, *P* < 0.001). **e** TrkB level in the mPFC (*F*_(3,20)_ = 0.340, *P* = 0.797). **f** Western blot bands of BDNF-TrkB signaling in the NAc. **g** BDNF level in the NAc (*F*_(3,20)_ = 17.414, *P* = 0.001). **h** proBDNF level in the NAc (*F*_(3,20)_ = 21.974, *P* < 0.001). **i** p-TrkB/TrkB ratio in the NAc (*F*_(3,20)_ = 10.828, *P* = 0.003). **j** TrkB level in the NAc (*F*_(3,20)_ = 0.140, *P* = 0.933). Data are shown as mean ± S.E.M. (*n* = 6). *BDNF* brain-derived neurotrophic factor, *mPFC* medial prefrontal cortex, *NAc* nucleus accumbens, *N.S*. not significant, *p-TrkB* phosphorylation-tropomyosin-receptor kinase B, *proBDNF* precursor of BDNF; *P < 0.05, **P < 0.01 or ***P < 0.001

To further elucidate the role of BDNF-TrkB signaling in the pathogenesis and therapeutic mechanisms of pain-induced anhedonia, we performed immunoblot analysis. Treatment with 7,8-DHF (10 mg/kg), but not ANA-12 (0.5 mg/kg), significantly improved abnormal levels of BDNF and proBDNF, and the ratio of p-TrkB/TrkB in the mPFC of anhedonia-like phenotype rats after SNI (Fig. 6a–e). In contrast, treatment of ANA-12, but not 7,8-DHF, significantly improved the abnormalities of BDNF-TrkB signaling in the NAc of anhedonia susceptible rats (Fig. 6f, j). However, there were no significant changes of total TrkB levels in the mPFC and NAc among the four groups (Fig. 6e, j). The data suggest that normalization of reduced BDNF-TrkB signaling in the mPFC by 7,8-DHF might play a role in its anti-anhedonia effects in the anhedonia susceptible rats.

## Discussion

Although SNI rats suffered almost identical nociceptive damage, only some rats exhibited anhedonia-like phenotypes. Tissue levels of BDNF in the mPFC in rats with anhedonia-like phenotypes were lower compared with those in rats without anhedonia-like phenotypes and sham-operated rats. In contrast, tissue levels of BDNF in the NAc in rats with anhedonia-like phenotypes were higher compared with those in rats without anhedonia-like phenotypes and sham-operated rats. Furthermore, tissue levels of BDNF in the hippocampus, L2–5 spinal cord, muscle, and liver of rats with or without anhedonia-like phenotypes were lower compared with those of sham rats. Interestingly, a single dose of the TrkB agonist 7,8-DHF (10 mg/kg) ameliorated anhedonia-like behaviors and decreased BDNF-TrkB signaling in the mPFC of the rats with anhedonia-like phenotypes after SNI surgery. To the best of our knowledge, this is the first study showing the role of BDNF-TrkB signaling in individual differences of anhedonia after neuropathic pain. This is also the first study demonstrating the role of BDNF-TrkB signaling in the beneficial effects of 7,8-DHF in the comorbidity of neuropathic pain and depression in rodents.

In preclinical studies, rats with hyperalgesia were highly heterogeneous in depression-related behaviors. Keay and colleagues reported that chronic constriction injury (CCI) induced a subgroup (approximately 30%) of rats that had altered dominant behavior [8] and sleep–wake cycles [38] using resident–intruder social interaction and sleep–wake analyses. In this study, the hierarchical cluster analysis divided SNI rats into two clusters: one group (approximately 40%, ‘anhedonia-like phenotype’) with reduced sucrose preference in the SPT, the other group (approximately 60%, ‘without anhedonia-like phenotype’) with similar sucrose preference compared with sham-operated rats. The previous clinical studies demonstrated that the incidence of comorbid chronic pain and depression is around 30–50% [1, 4, 5], which is in line with the present study. In this study, we found that rats with or without anhedonia-like phenotypes showed similar mechanical withdrawal thresholds, suggesting that the alterations in mood-related behaviors were independent of the degree of nociceptive damage, consistent with the previous studies [8, 31, 38–40].

We found decreased BDNF-TrkB signaling in the mPFC and hippocampus from rats with anhedonia-like phenotypes on day 23 after SNI, consistent with the previous study [31]. Low levels of BDNF in the mPFC are associated with the development of depression-like phenotypes including anhedonia in rodents [19–28, 41]. In contrast, we found increased BDNF-TrkB signaling in the NAc from rats with anhedonia-like phenotypes, consistent with the previous reports from rodents with depression-like phenotypes [19–28]. Interestingly, ANA-12 did not improve the decreased sucrose preference of rats with anhedonia-like phenotypes, although ANA-12 significantly attenuated increased BDNF-TrkB signaling in the NAc from rats with anhedonia-like phenotypes. Thus, it is unlikely that BDNF-TrkB signaling in the NAc plays a role in the anhedonia-like phenotype after SNI. Taken together, it is likely that decreased BDNF-TrkB signaling in the mPFC might be implicated in the anhedonia-like phenotype in rats with neuropathic pain, and that TrkB agonists are potential therapeutic drugs for anhedonia in patients with neuropathic pain.

In this study, we found that both SNI rats with or without anhedonia-like phenotypes have lower tissue levels of BDNF in the L2–5 spinal cord compared to sham-operated rats. Given the role of BDNF-TrkB signaling in pain, it seems that reduced BDNF-TrkB signaling in the L2–5 spinal cord may play a role in the neuropathic pain, although BDNF-TrkB signaling in this region may not play a role in the anhedonia-like phenotype.

As well as the brain, expression of proBDNF, BDNF, and its receptor TrkB has been reported in the liver and muscle [42–45], although the functional role of proBDNF-BDNF-TrkB signaling in these tissues is not fully understood. The high expression of BDNF and TrkB proteins in the livers suggests neurotrophic support for autonomic innervation of this organ [43, 44]. A recent study suggests the role of BDNF-TrkB signaling in the synapse maintenance and function in the neuromuscular system [46]. Nonetheless, further detailed studies of the underlying role of proBDNF-BDNF-TrkB signaling in these organs are needed. In addition, we also found both SNI rats with or without anhedonia-like phenotypes have lower tissue levels of BDNF in the muscle and liver compared with those in sham-operated rats. Interestingly, we observed alterations in BDNF-TrkB signaling in the liver from mood disorders including major depressive disorder and bipolar disorder, suggesting a possible role of BDNF-TrkB signaling in mood disorders [47]. Given the key role of BDNF-TrkB signaling in psychiatric disorders [48, 49], it is likely that alterations in BDNF-TrkB signaling may contribute to anhedonia susceptibility to SNI surgery. Further detailed studies on the role of BDNF-TrkB signaling in the brain as well as peripheral organs in the SNI model are needed.

In conclusion, the current study suggests that decreased BDNF-TrkB signaling in the mPFC plays key roles in individual differences of the anhedonia-like phenotype in rats with neuropathic pain. Therefore, it is likely that activation at BDNF-TrkB signaling could be a potential therapeutic target for anhedonia in patients with neuropathic pain.
